# Use of Counter-Irritants

**Published:** 1868-05

**Authors:** 


### Use of Counter-Irritants.

Prof. Hebra, the celebrated dermatologist, declares his opposition to the use of counter-irritants (revulsives,) which form so large a part of ordinary medical practice. He professes to show that the theory by which their use was originally suggested and is now maintained, is erroneous; and he brings forward clinical and experimental facts and arguments to demonstrate the evils which result from the practice. Physicians were, he maintains, led to adopt counter-irritants for the cure of internal diseases, by observing the alterations and apparent antagonism of cutaneous affections and internal lesions. When, for example, a patient, the subject of a skin disease (as psoriasis,) was attacked by fever, the chronic cutaneous eruption was observed to fade and disappear while the febrile state lasted, but re-appeared during convalescence. Again, in exanthematous fevers the febrile symptoms often subside on the appearance of the specific eruption; and, moreover, the eruption continues fully out only under a favorable condition of the internal affection; while, on the contrary, if the internal symptoms are aggravated, the cutaneous eruption diminishes, and, before a fatal issue, becomes altogether invisible. Hence the popular idea among physicians and the laity, that the suppression or metastasis of the cutaneous affection is the cause of the fatal issue of the general disease. But these opinions are erroneous, for the chronic skin disease does not vanish first, to be replaced by the fever; but, on the contrary, the dermatosis only begins to fade after a prolonged continuance of an intense febrile state. In like manner, in every general disease of great intensity and prolonged duration, the anæmia which supervenes is first noticed on the skin, and hence the fading and disappearance of red eruptions in these cases, since even syncope will cause their temporary vanishing. Another erroneous opinion, prevalent from ancient times till the present day, which probably lent support to the use of counter-irritants, was the idea that every disease consisted of something material, which attacked sometimes one part, sometimes another, and which it was the physician's chief aim to eliminate; hence the terms *materia peccans*, acrimonies of the blood, acids, phlegm, black and yellow bile, etc. These notions, in spite of modern pathology, still retain their influence on practice. It is, however, certain, that we cannot judge of the use of remedies on such grounds, but only by a knowledge of the action of the remedy on the healthy body on the one hand, and an acquaintance with the natural course of disease, uninfluenced by remedies, on the other. This was first stated by Gideon Harvey, in his work, "Ars Curandi Morbos Expectatione, Amstelodami, 1695," where he says boldly that "it would be proper to write on the patient's prescription only the word, *Expecta.*" But this expectant method seemed a neglect too cruel to be practiced in dangerous diseases, until Hahnemann showed that fevers and inflammations were often as successfully treated by decillionths as by the Hippocratic apparatus of venesection and counter-irritation; whereupon physicians and clinical professors, convinced of the real nothingness of decillionths, resorted to the treatment of febrile and non-febrile diseases by the pure expectant method, and were thus enabled to study accurately the natural course of disease. Whereever their results were as successful as the older heroic practice pursued "lege artis," it was obvious that the latter was at least unnecessary, that the cure was done by nature in spite of heroic remedies: "Natura et morbum et medicum vincit." Nevertheless, some practitioners, who admit all this in regard to certain diseases, plead for counter-irritants to the skin in the class of affections called *rheumatism*, believing that the peripheral irritation will relieve the deeper seated parts. But how often do these remedies, from simple rubefacients up to the "horrible invention of the moxa," fail; so that where cases improve, it is rather *during* than *by* the treatment. An impartial examination would show that as many rheumatic affections get well under homœopathy hydropathy, electricity, or the plasters of quacks, as under counter-irritants.

Moreover, the excuse that it is necessary to do something to relieve suffering, and that blisters, etc., do no harm, is incorrect, as they often leave indelible marks on the skin, while hot and cold douches, lotions, liniments, and plasters do not, and yet afford as much relief. In diseases of the eye, also, the applications of leeches and blisters, which were formerly always used, are now condemned by many ophthalmologists. Clinical experiments in Hebra's practice gave similar evidence. Suppose on the thigh of a patient an eczema rubrum of the size of a crown-piece; place at two inches distance on one side a blister of the same size, on the opposite side a sinapism, and at the two other poles, tartar emetic ointment and croton oil. The artificial irritants will produce here bullæ and redness, there pustules and vesicles, but without at all diminishing the intensity of the central eczema. On the contrary, the latter often spreads to and includes the irritated surfaces, and becomes larger than before. Now, if in the same organ or tissue (the skin) a peripheral irritant cannot draw away and dispel a central affection of a similar kind, how can cutaneous irritation be expected to dissipate the morbid condition of the pleura, lungs, brain, peritoneum, eyes, sheaths of nerves, etc.? What new path do revulsives open up for the elimination of morbid products deposited in these cavities and organs? But cutaneous irritants, continues Prof. Hebra, are not only useless, but often do harm, and their pernicious effects may either last long, or even put life in peril. It is, for instance, a well known fact that the exanthemata, small-pox, measles, and scarlet fever, are more fatal in proportion to the intensity and amount of the cutaneous eruption. And experience teaches that the eruption is more abundant on parts of skin where an irritation or dermatosis (e. g., an eczema) previously existed. A sinapism, applied to the chest on account of dyspnœa, will cause a larger amount of pocks to appear there, and if there were a counter-irritant large enough to cover the entire integument, a simple varicella might be converted into a fatal variola. Indeed, even the irritation of cold water in excess, applied hydropathically around an eczema, will cause its extension over the whole skin; and often the abuse of acaricidal remedies—e. g., the sulphur fumigations in vogue a few years ago—produces a horrible eczema over the whole external surface; often hot baths, pushed by some as an infallible remedy, diffuse instead of arresting a skin disease. The tartar emetic ointment, applied to the scalp in chronic hydrocephalus, causes no diminution of the effusion, while the pustular eruption is very painful, and may give rise to purulent absorption and erysipelas. A blister behind the ear is often the starting point of an eczema which affects the concha, the face, and hairy scalp, and which, under unsuitable treatment, may last for years, causing great pain, as Hebra has often seen, without improving an ophthalmia in the least. Leech bites on the temples are followed by incurable white triangular cicatrices, which certainly are no ornament to a pretty face. The marks of cupping, too, on the neck and arms of women, are often a serious disfiguration. Issues in the arm to prevent relapses of ophthalmia, or of cerebral congestions, or as derivatives from skin diseases, are not only useless for their intended purposes, but are very troublesome and frequently become the source of eczema which spread over the surrounding integument. Even the discolorations which sinapisms leave behind, entail a lasting defect when applied to the neck or chest in females. Sometimes, indeed, a fatal result may be caused through the application of counterirritants in typhoid fever, pneumonia, or small-pox, in consequence of the blistered surface being the seat of *cutaneous diphtheria.* The tinct. of arnica, introduced by homœopathists, if diluted, as they use it, is harmless; but if applied in a concentrated form, it causes redness and swelling of the skin, which in sensitive individuals develops into an eczema which spreads over a large surface and even the whole skin, and requires months to heal, confining the patient to bed for a longer period and a worse condition than the affection would have done for which the arnica was administered. Many of these artificial skin eruptions, bullous, vesicular, and pustular, do not cease when the counter-irritant which caused them is removed, but last for weeks, months, or years. They can then no longer be distinguished from the idiopathic skin diseases, eczema, pemphigus, ecthyma, impetigo; and were the common theory true of the protective influence of counter-irritation, these skin diseases ought to afford a security against internal diseases in proportion to their amount. In that case, no persons should be healthier than the subjects of general chronic pemphigus, which should afford means of elimenating all kinds of peccant matters from the system. On the contrary, however, experience teaches that exudative eruptions covering large surfaces, especially in the form of vesicles, blebs, or pustules, not only exhibit no preservative power, but the reverse, exerting a disastrous influence on the general health, and becoming a frequent source of fatal issues. *Lazarus* was always regarded as a collection of all kinds of diseases, requiring providential intervention for his cure! Veterinary art exhibited similar prejudices till a few years ago. Diseased animals, especially horses, used to be tortured with all kinds of cauteries and corrosives; and farriers were in repute with the ignorant in proportion to the cruelty of their treatment. But thanks to the heads of the medical department in the Veterinary Institute of Vienna, such practice is now obsolete there, and results equally satisfactory are now obtained by the expectant method as were formerly got by those cruel manœuvres. Veterinarians, in this respect, according to Hebra, might serve as a model for physicians. "It would be desirable," he says in conclusion, "that every physician, before applying a counter-irritant to his patient, should ask himself the question, whether, if he were ill, he would treat himself in the same way? Indeed, it has rarely happened to me to see physicians who would have subjected their own persons to the application of issues, moxas, and setons. Yet, let not the physician forget the commandment, 'Do not that to others which you would not have done to yourself,' when, at the bedside of his patient, he finds other remedies unavailing, and is tempted to have recourse to epispastics as his last resort. He ought always to remember that his mission is to relieve pain, and whereever his attempts to do so fail in spite of all his efforts, he can console himself with the assurance that he has at least caused no superfluous suffering."—*Edinburgh Med. Journal,* Nov., 1867, from *Allg. Med. Zeit.*

